# Gut Microbiota α- and β-Diversity, but Not Dietary Patterns, Differ Between Underweight and Normal-Weight Japanese Women Aged 20–39 Years

**DOI:** 10.3390/nu17203265

**Published:** 2025-10-17

**Authors:** Risako Yamamoto-Wada, Eri Hiraiwa, Kana Okuma, Masako Yamada, Chihiro Ushiroda, Kanako Deguchi, Hiroyuki Naruse, Hiroaki Masuyama, Katsumi Iizuka

**Affiliations:** 1 Department of Clinical Nutrition, Fujita Health University, Toyoake 470-1192, Japan; risako.wada@fujita-hu.ac.jp (R.Y.-W.); 51022099@fujita-hu.ac.jp (E.H.); chihiro.ushiroda@fujita-hu.ac.jp (C.U.); kanasakuran@gmail.com (K.D.); 2 Faculty of Medicine, Fujita Health University, Toyoake 470-1192, Japan; 3 Symbiosis Solutions Inc., Tokyo 101-0064, Japan; okuma@symbiosis-solutions.co.jp (K.O.); yamada.m@symbiosis-solutions.co.jp (M.Y.); masuyama@symbiosis-solutions.co.jp (H.M.); 4 Health Management Center, Fujita Health University, Toyoake 470-1192, Japan; hnaruse@fujita-hu.ac.jp; 5 Food and Nutrition Service Department, Fujita Health University Hospital, Toyoake 470-1192, Japan

**Keywords:** underweight, gut microbiota, α-diversity, β-diversity, dietary pattern

## Abstract

**Background and Aim:** Underweight young adult women are vulnerable to health risks such as menstrual disorders and vitamin deficiencies. Because few seek medical care for low body weight, the underlying causes remain unclear. This study aimed to examine the associations of body type with dietary patterns and gut microbiota diversity in young women. **Methods:** We enrolled 40 women aged 20–39 years who visited a nutrition evaluation clinic with a BMI < 17.5 at their first consultation (underweight group) and 40 age-matched women with 18.5 ≤ BMI < 25 (control group). Some women in the underweight group were no longer underweight at the time of analysis but were classified based on their initial BMI. Dietary patterns were assessed based on ten major food categories (meat, fish, eggs, dairy products, soybeans, green and yellow vegetables, seaweed, fruit, tubers, and fats and oil) based on the Food Frequency Questionnaire based on Food Groups. Gut microbiota α-diversity was evaluated using the Shannon, Simpson, and Pielou indices, while β-diversity was analyzed by nonmetric multidimensional scaling (NMDS) and redundancy analysis (RDA). Genera contributing to group differences were identified by RDA and ANOVA-Like Differential Expression tool (ALDEx2). **Results:** Underweight women had significantly lower gut microbiota α-diversity, while no difference was observed in dietary patterns. NMDS revealed significant β-diversity differences in gut microbiota (PERMANOVA: R^2^ = 0.064, F = 5.31, *p* = 0.0001) but not in dietary patterns (*p* = 0.99). RDA showed that body type explained 4.5% of variance (adjusted R^2^ = 0.032, F = 3.65, *p* = 0.0005). *Bacteroides*, *Bifidobacterium*, *Enterocloster*, and *Erysipelatoclostridium* were enriched in underweight women, whereas *Fusicatenibacter*, *Agathobacter*, *Dorea*, and *Prevotella* were enriched in controls. AldEx2 confirmed increases in *Bacteroides*, *Enterocloster*, and *Erysipelatoclostridium* and a decrease in Dorea. **Conclusions:** Underweight women demonstrated reduced gut microbiota diversity and enrichment of taxa associated with inflammatory tendencies. Dietary therapies involving not only prebiotics but also probiotics may beneficially modulate gut microbiota and contribute to the management of low body weight.

## 1. Introduction

Lean body weight in young women has been associated not only with menstrual irregularities and infertility but also with decreased immune function and future declines in bone density [1,2,3,4]. In Japan, the proportion of underweight women is greater than that in Western countries, with a notably high percentage—20%—of Japanese women between the ages of 20 and 39 classified as underweight [5,6]. The proportion of underweight women aged 20 to 39 in Japan has not changed for the past 20 years. To clarify the reality of underweight women in Japan, we conducted nutritional assessment consultations for individuals with a BMI of less than 17.5 who had undergone staff health checkups [7]. We reported that a certain number of these individuals presented decreased grip strength, a greater frequency of vitamin deficiencies, lower levels of nutritional markers such as cholesterol and lymphocytes, and reduced prealbumin levels [7,8]. Additionally, the dietary patterns of women aged 20–29 years differ markedly from those of men in the same age group, and women’s dietary patterns change and become more diverse with age [9]. In other words, the results showed that for women in their 40s and 50s, fruits, milk, and seaweed contributed to the formation of dietary patterns, while for women in their 20s and 30s, meat and eggs played a contributory role [9]. According to the 2001–2019 National Health and Nutrition Survey in Japan, too, a decreased trend in fish, shellfish, and seaweed intake and an increased trend in meat and soft drink intake were observed among young women aged 20–39 years [10]. Decreased trends in the intake of fruit and dairy products were observed in young women who were not obese [10]. Regarding dietary characteristics of underweight women aged 20–39 years, 32% of the underweight women aged 20–39 years in this study skipped breakfast, and 50% had low dietary diversity scores [7]. However, whether dietary patterns vary according to body type within the same age group has not yet been clarified. In addition to problems with dietary habits, diseases may also be causes of weight loss. Low body weight in women can be caused by conditions such as Graves’ disease, adrenal insufficiency, and cancer [6], so it is necessary to differentiate among the causes of low body weight [7]; however, opportunities for medical treatment of idiopathic underweight remain limited.

In addition to dietary patterns, gut microbiota patterns are also correlated with body composition [11,12,13,14,15,16]. An association between the gut microbiota and obesity has been reported, with the abundance of *Firmicutes* tending to increase and that of *Bacteroidetes* tending to decrease in more obese patients [11]. Similarly, the gut microbiota patterns of underweight individuals reportedly differ from those of people with normal weight [15]. In patients with eating disorders, such as extremely underweight patients with anorexia nervosa (AN), data indicate a decrease in the abundance of butyrate-producing bacteria (such as *Roseburia* and *Clostridium*) [16]. Comparisons of the gut microbiota and intestinal metabolites in normal weight, overweight, or underweight adults revealed lower alpha diversity among those at either extreme (overweight and underweight) [16]. However, the differences in the diversity of the gut microbiota of young Japanese women by body weight have not been investigated. If differences in the gut microbiota between thin young women and women of normal weight become clear, I believe that improving eating habits could lead to improvements in the gut microbiota and, consequently, body weight.

In this study, we compared the diversity of dietary patterns and gut microbiota patterns between young adult underweight women and normal weight women and clarified whether changes in the gut microbiota are involved in the mechanisms that lead to underweight. Specifically, we compared dietary diversity and gut microbiota patterns between underweight and normal-weight women aged 20–39 years. By identifying characteristic bacterial taxa in underweight individuals, we aim to provide insights that may contribute to future dietary interventions involving not only prebiotics but also probiotics, as well as to a better understanding of the mechanisms underlying gut microbiota alterations in underweight individuals.

## 2. Materials and Methods

### 2.1. Study Design

This study was conducted as a cross-sectional observational study to compare the dietary pattern and gut microbiota profiles between normal weight and underweight groups in Japanese young adult women aged 20–39 years. The research participants were young female staff members of Fujita health university hospital who were referred from the health management department to the nutrition assessment outpatient clinic for a BMI less than 17.5 and who were actively receiving regular outpatient care. Fujita Medical University is located near Nagoya, one of Japan’s major metropolitan areas. The surrounding area, however, also includes agricultural fields and open spaces, illustrating the typical coexistence of urban and rural environments found throughout much of Japan. We recruited research participants from among those visiting the clinic who wished to have their gut microbiota analyzed. Recruitment of participants was conducted from March 2025 to May 2025. We explained the study to 45 individuals and invited them to participate. Among the invited participants, 44 signed an informed consent form and were enrolled in the study; of these 44, only 40 were sent samples within one month after receiving the kit at the clinic. In this study, the samples from 40 individuals (32 in their 20s and 8 in their 30s) whose stool samples were obtained via symbiosis solutions were categorized into the low-body-weight group.

For the normal weight group, 40 women (32 in their 20s and 8 in their 30s) were randomly selected from among 127 women aged 20–40 years with normal weight and who had not been flagged for any abnormalities during screening held by Symbiosis Solutions Co., Ltd. (Tokyo, Japan).

The extraction criterion for participants was Japanese women aged 20–39 years with a BMI between 18.5 and 25. The exclusion criteria included taking oral medications, having an existing illness, being pregnant, and collecting stool samples from enemas or artificial stomas. Except for BMI, these conditions were common to all the study participants.

We published on the websites of the Department of Clinical Nutrition at Fujita Health University and Symbiosis Solutions Co., Ltd., in Japan, and unlinked data from normal weight individuals were used as controls for this study. The study was conducted in accordance with the principles of the Declaration of Helsinki and approved by the Research Ethics Committee of Fujita Health University (application number: HM24-390; approval date: 4 February 2025).

### 2.2. Data Collection

With respect to body weight, after their weight was measured during the medical examination, the participants completed the questionnaire along with their height. The collected data included participant height, weight, and age and the results of the FFQg (Food Frequency Questionnaire based on Food Groups) dietary survey (energy, carbohydrates, fats, protein, dietary fiber) [9,17,18]. A food frequency questionnaire (FFQg) was developed, based on 29 food groups and 10 kinds of cookery, for estimating the energy and nutrient intakes of an individual subject during the previous one to two months [17]. The FFQ asks about the frequency of consumption and the amount used per serving of each food, and is a method for estimating individual energy and nutrient intake. The 29 food groups include rice, bread, noodles, meat and processed meats, seafood, eggs, soybeans, dairy products, seafood, seaweed, small fish, green and yellow vegetables, mushrooms, fruits, jams and honey, among others. The FFQg is among the most widely used food intake frequency questionnaires in Japan [6,9,17,18] and includes questions on the frequency of the consumption of 10 different food types (meat, fish, eggs, dairy products, soybeans, green and yellow vegetables, seaweed, fruit, tubers, and fats and oil) over seven days [9].

### 2.3. DNA Extraction and Sequencing

DNA extraction from stool samples and 16S rRNA sequencing were performed via methods previously reported by Kono et al. [19]. Briefly, DNA was extracted via an automated enterobacterial DNA extractor (DEX-I; PMT Corp., Fukuoka, Japan) with lysozyme, glass beads, and GTC solution, followed by phenol–chloroform extraction and ethanol precipitation. The DNA was stored at −80 °C until use.

### 2.4. PCR Amplification

The V3–V4 regions of the 16S rRNA gene were amplified from 12.5 ng of extracted DNA via Q5 Hot Start High-Fidelity DNA Polymerase (New England Biolabs, Ipswich, MA, USA), as previously described [19]. The PCR products were subsequently purified with AMPure XP beads (Beckman Coulter, Brea, CA, USA).

### 2.5. Library Preparation and Sequencing

Indexed libraries were prepared with the Nextera XT Index Kit v2 (Illumina, San Diego, CA, USA) and quantified via the QuantiFluor dsDNA System (Promega, Madison, WI, USA). Libraries were diluted to 4 nM, pooled with the PhiX control, and sequenced on an Illumina MiSeq platform via the Reagent Kit v3 (2 × 300 cycles).

### 2.6. Data Processing and Analysis

Raw sequences were processed with bcl2fastq (Illumina) and clsplitseq (ver. 0.2.2019.05.10) for demultiplexing and primer removal. Quality filtering and ASV inference were performed with DADA2 (ver. 1.16) in R (ver. 4.0.3). Rarefaction was conducted via the vegan package (ver. 2.5.7), and taxonomy was assigned to the RDP training set 18.

### 2.7. Data Preprocessing

Genus-level gut microbiota data were used for multivariate analyses. The raw frequency data were transformed into relative abundances by dividing each entry by the row sum to account for variation in the sampling depth. For the RDA, the relative abundances were further subjected to Hellinger transformation to reduce the influence of highly abundant taxa. All analyses were performed in R (version 4.5.1) via the vegan (version 2.7-1), ggplot2 (version 4.0.0), and ggrepel packages (version 0.9.6).

### 2.8. Statistical Analysis

#### 2.8.1. Alpha Diversity Indices

Dietary alpha diversity was assessed via the Shannon, Simpson, and Pielou indices, which were calculated for each participant. Differences in BMI between the two groups (underweight and control groups) were analyzed. The Shannon diversity index for each sample was calculated in Excel. Alpha diversity was evaluated via the Shannon (H′ = −∑piln(pi)), Simpson (1 − ∑pi2), and Pielou (J′ = H′/lnS) indices. Dietary patterns were calculated on the basis of the frequency of consumption of ten food items over seven days [9]. Gut microbiota patterns were also calculated from the genus counts obtained via high-speed sequencing. Group comparisons were performed via nonparametric tests. For two-group comparisons, the Mann–Whitney U test was applied to assess differences between groups. To evaluate the magnitude of the differences, Cliff’s delta was calculated along with its 95% confidence interval. The interpretation of Cliff’s delta followed the guidelines of Vargha and Delaney (2000) [20], where |δ| < 0.147 indicates no effect, 0.147 ≤ |δ| < 0.33 indicates a small effect, 0.33 ≤ |δ| < 0.474 indicates a medium effect, and |δ| ≥ 0.474 indicates a large effect. A two-tailed *p* value < 0.05 was considered to indicate statistical significance [20].

To evaluate the associations between specific parameters of interest (body mass index (BMI), energy intake, protein intake, carbohydrate intake, lipid intake, and dietary fiber intake) and diversity indices (the Shannon index of the gut microbiota and the dietary Shannon index), nonparametric correlation analyses were conducted. Spearman’s rank correlation coefficient (ρ) was calculated along with the *p* values. All analyses were performed via R software (version 4.5.1, R Foundation for Statistical Computing, Vienna, Austria). Visualization was carried out with the ggplot2 package, and scatterplots were generated with LOESS smoothing. Spearman’s ρ and corresponding *p* values are annotated on the plots. The strength of Spearman’s rank correlation coefficient (ρ) was interpreted as negligible (<0.1), weak (0.1–0.3), moderate (0.3–0.5), or strong (≥0.5), according to commonly used guidelines. A two-tailed *p* value < 0.05 was considered to indicate statistical significance.

#### 2.8.2. Nonmetric Multidimensional Scaling (NMDS)

To evaluate overall differences in community composition between groups (normal weight group (C: control) vs. underweight group (L: lean)), nonmetric multidimensional scaling (NMDS) was performed on the basis of Bray–Curtis dissimilarities via the metaMDS function (vegan package). Ordination was conducted with two dimensions (k = 2), and up to 200 iterations were allowed for convergence. The beta diversity of the gut microbiota was assessed via NMDS on the basis of Bray–Curtis dissimilarities. Group differences were tested via permutational multivariate analysis of variance (PERMANOVA) with 9999 permutations, and the homogeneity of multivariate dispersion was evaluated via Permutational Analysis of Multivariate Dispersions (PERMDISP). To assess the robustness of the group centroids, bootstrap resampling (B = 2000) was applied to calculate 95% confidence intervals. For all the statistical analyses, two-tailed *p* values < 0.05 were considered to indicate statistical significance. All analyses were performed in R (version 4.5.1, R Foundation for Statistical Computing) via the vegan and ggplot2 packages.

#### 2.8.3. Redundancy Analysis (RDA)

Redundancy analysis (RDA) was performed only for gut microbiota patterns that showed significant group differences in the NMDS analyses. Genus-level data were Hellinger-transformed and analyzed via the rda function (vegan package, version 2.7-1), with group (C vs. L) as the explanatory variable. The model summary parameters included total inertia, coefficient of determination (R^2^ and adjusted R^2^), variance explained by each constrained axis, and permutation test results (overall model, axes, and terms; 9999 permutations). Significantly constrained axes and explanatory terms (*p* < 0.05) were identified. The group centroids and their 95% confidence intervals were estimated via bootstrap resampling (B = 2000). Species scores (scaling = 2) were used to quantify taxon contributions, and the top 10 taxa were visualized in biplots (RDA1-only).

#### 2.8.4. ANOVA-like Differential Expression Tool, Version 2 (AldEx2)

In RDA, taxon contributions to constrained axes are evaluated on the basis of species scores, but the statistical significance of individual taxa cannot be formally tested within this framework. Therefore, differential abundance analysis was additionally performed via ALDEx2 (ANOVA-Like Differential Expression tool, version 2) to identify taxa showing statistically significant differences between groups [21,22,23]. Differential abundance analysis was performed via ALDEx2 (v1.40.0, R v4.5.1), which applies centered log-ratio (CLR) transformation with 2000 Monte Carlo samples from the Dirichlet distribution to account for compositionality and technical variation. Group comparisons (C vs. L) were tested via Welch’s *t* test and the Wilcoxon rank-sum test with Benjamini–Hochberg correction for multiple testing. Effect sizes were estimated, and taxa with FDR-adjusted *p* values < 0.05 were considered significant. The results were visualized in volcano plots (effect size vs. −log10[FDR]) generated via ggplot2 with highlighted taxa of interest.

## 3. Results

### 3.1. Participant Characteristics

First, I describe the background of the research participants. A comparison was conducted between 40 underweight individuals and 40 controls. There was no significant difference in the mean age between the normal weight (C: control) group (n = 40) and the underweight (L: lean) group (n = 40) (27.88 vs. 27.40, *p* = 0.65). In contrast, BMI differed significantly between the two groups (20.77 (1.41) vs. 17.07 (0.97)). No differences were observed in energy, fat, protein, carbohydrate, or dietary fiber intake (*p* = 0.48, *p* = 0.74, *p* = 0.73, *p* = 0.57, and *p* = 0.87, respectively) (Table 1). In addition to body weight, no significant differences were observed between the two groups.

### 3.2. Alpha Diversity of Dietary and Gut Microbiota Patterns

**Figure 1:**
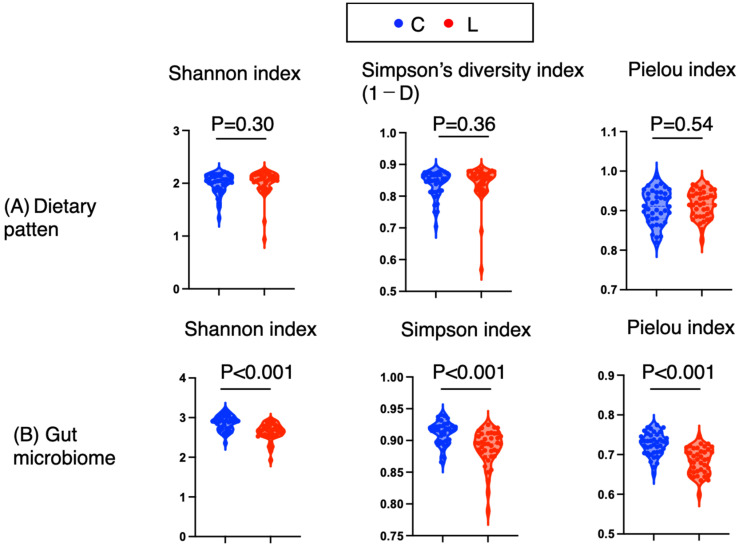
Alpha diversity indices of dietary and gut microbiota patterns. (**A**) The alpha diversity of the dietary pattern was evaluated for the incidence of 10-item food intake per week via the Shannon (H′ = −∑piln·pi ), Simpson (1 − ∑pi2), and Pielou (J′ = H′/lnS) indices. (**B**) The alpha diversity of the gut microbiota was evaluated from genus-level counts obtained via high-throughput sequencing via the Shannon (H′ = −∑pilnpi), Simpson (1 − ∑pi2), and Pielou (J′ = H′/lnS) indices. C: normal weight group; L: underweight group. *p* < 0.05 is significant.

α diversity is an index that reflects the diversity within an individual; the α diversity of dietary patterns indicates whether a person consumes a variety of foods. Using the Shannon, Simpson, and Pielou indices, we compared the diversity of dietary patterns and gut microbiota patterns between groups. For dietary patterns, no significant differences were found in the Shannon, Simpson, or Pielou indices (*p* = 0.30, *p* = 0.36, and *p* = 0.54, respectively), and Cliff’s delta values were negligible [Cliff’s δ: −0.13, 0.12, and −0.079, respectively] (Figure 1A and Table 2). Thus, the α diversity of the dietary patterns was similar between the two groups.

In contrast, the α diversity of the gut microbiota reflects the types and balance of gut bacteria. The gut microbiota diversity differed significantly between the groups. The Shannon, Simpson diversity, and Pielou indices were significantly greater in the control group than in the low-body-weight group (*p* < 0.001, *p* < 0.001, and *p* < 0.001, respectively), and the Cliff delta values exceeded 0.5, indicating large effects [0.59, 0.55, and 0.67, respectively] (Figure 1B and Table 2). Thus, the α diversity of the gut microbiota in the control groups was significantly greater than that in the underweight groups.

### 3.3. Correlations of Alpha Diversity with BMI and Nutrient Intake

**Figure 2:**
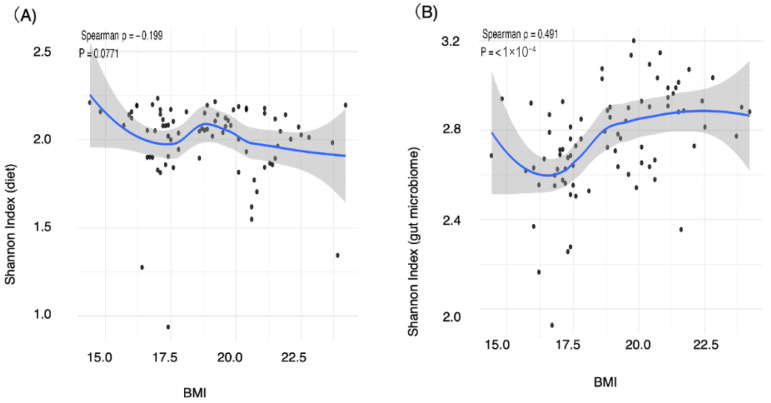
Alpha diversity of dietary and gut microbiota patterns. (**A**) Shannon index of dietary patterns (**B**) Shannon index of the gut microbiota patterns. To evaluate the associations between BMI and the Shannon index, nonparametric correlation analyses were conducted. Spearman’s rank correlation coefficient (ρ) was calculated along with the *p* values. Visualization was carried out with the ggplot2 package, and scatterplots were generated with LOESS smoothing. Spearman’s ρ and corresponding *p* values are annotated on the plots. (**A**) Spearman ρ = −0.19, *p* = 0.077, (**B**) Spearman ρ = 0.49, *p* < 0.0001.

We next examined the correlations between the Shannon indices of the dietary patterns and the gut microbiota and variables such as BMI, age, and nutrient intake via Spearman’s rho. The Shannon index of dietary patterns showed a bell-shaped association with BMI, peaking around a BMI of 19, but there was no significant correlation overall (ρ = −0.19, *p* = 0.077) (Figure 2A, Table 3). The Shannon index exhibited strong positive correlations with protein intake (ρ = 0.4, *p* < 0.0001) and dietary fiber intake (ρ = 0.63, *p* < 0.0001) (Table 3).

With respect to the gut microbiota, the Shannon index increased steadily as BMI increased from 16 to 19 and continued to increase gradually thereafter, indicating a significant positive correlation with BMI (ρ = 0.49, *p* < 0.0001) (Figure 2B, Table 3). No correlations were detected between the Shannon index and age, total energy intake, carbohydrate intake, protein intake, fat intake, or dietary fiber intake (*p* = 0.97, *p* = 0.56, *p* = 0.37, *p* = 0.36, *p* = 0.81, and *p* = 0.24, respectively) (Table 3). Thus, the Shannon index of dietary patterns correlated strongly with protein and dietary fiber intake, whereas the Shannon index of gut microbiota patterns correlated strongly with BMI. C: normal weight group; L: underweight group.

### 3.4. The Beta Diversity of Gut Microbiota Patterns Rather Than Dietary Patterns Differed Between the Normal and Underweight Groups

Since significant differences in the Shannon diversity of the gut microbiota were observed between the normal and underweight groups, we further examined whether the overall community composition differed between the groups. For this purpose, β diversity analyses were performed. First, NMDS was applied to visualize the natural clustering of samples.

**Figure 3:**
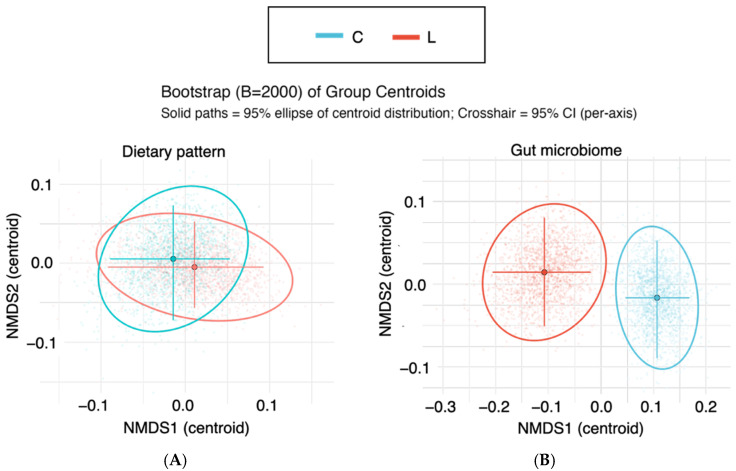
Nonmetric multidimensional scaling of dietary patterns and gut microbiota patterns. Nonmetric multidimensional scaling (NMDS) ordination of (**A**) dietary patterns and (**B**) gut microbiota composition on the basis of Bray–Curtis dissimilarity. Group centroids were estimated from 2000 bootstrap resamplings, and bias-corrected and accelerated (BCa) 95% confidence intervals are depicted as ellipses around the centroids. The ellipses represent the variability of the bootstrap distribution, not the confidence ellipses of the raw data points. C: normal weight group; L: underweight group.

First, regarding dietary patterns, no significant difference in group centroids was observed between the C and L groups (PERMANOVA: R^2^ ≈ 0, F ≈ 0, *p* = 0.99). Similarly, there was no significant difference in within-group dispersion (PERMDISP: F = 0.44, *p* = 0.52) (Figure 3A, Table 4). Thus, no differences were observed between the two groups in their eating patterns.

In contrast, β diversity analysis of the gut microbiota revealed a significant difference in community composition between the C and L groups (PERMANOVA: R^2^ = 0.064, F = 5.31, *p* = 0.0001). Although no significant difference in dispersion was detected (PERMDISP: F = 3.21, *p* = 0.072), the distance to the centroid was slightly greater in the L group, suggesting a tendency toward greater variability. However, the 95% confidence intervals overlapped between the groups (Figure 3B, Table 4). Therefore, differences were observed in the gut microbiota patterns between the two groups.

### 3.5. The Difference in Bacterial Composition Between the Normal and Underweight Groups Reflects the Beta Diversity of the Gut Microbiota

RDA is a constrained ordination method that incorporates explanatory variables to statistically model and test whether factors such as BMI group (C vs. L) influence community composition. No significant differences in dietary patterns were detected between the groups; however, the centroids of the gut microbiota patterns differed significantly between the C and L groups. Therefore, RDA was performed only for the gut microbiota patterns, using BMI category (C vs. L) as the explanatory variable.

The total variance of the dataset was 0.280, of which approximately 4.5% was explained by the explanatory variable (adjusted R^2^ = 0.032). The overall model was significant according to permutation testing (F = 3.65, *p* = 0.0005). Among the constrained axes, RDA1 accounted for 4.5% of the total variance and was statistically significant (*p* = 0.0005) (Table 5). Therefore, 4.5% of this RDA model was explained by body constitution (normal weight vs. underweight).

**Figure 4:**
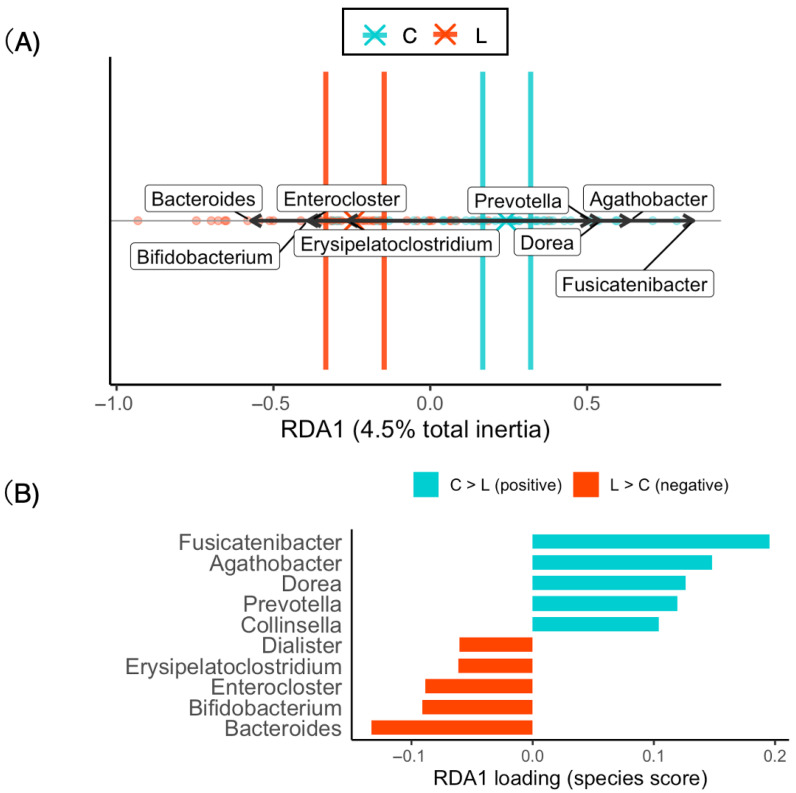
Redundancy analysis (RDA) of the gut microbiota composition. (**A**) RDA biplot showing separation between the control (C, normal weight) and lean (L, underweight) groups. The ellipses represent 95% confidence intervals obtained via bootstrap resampling. The arrows indicate taxa contributing most strongly to group separation, with *Fusicatenibacter*, *Agathobacter*, *Dorea*, and *Prevotella* associated with the positive side of RDA1 and *Erysipelatoclostridium*, *Enterocloster*, *Bacteroides*, and *Bifidobacterium* associated with the opposite side. (**B**) Bar plot showing the top 10 taxa contributing to RDA1, ordered by effect size. C: normal weight group; L: underweight group.

The RDA biplot (Figure 4A) clearly shows separation between groups, with bootstrap-based confidence ellipses indicating distinct clustering. The arrows representing the major contributing taxa are oriented along the axis of group separation. The taxa most strongly associated with the positive side of RDA1 included *Fusicatenibacter*, *Agathobacter*, *Dorea*, and *Prevotella*, whereas *Erysipelatoclostridium*, *Enterocloster*, *Bacteroides*, and *Bifidobacterium* contributed in the opposite direction (Figure 4A). The top 10 taxa contributing to RDA1 are summarized in Figure 4B.

### 3.6. Differential Abundance Analysis by ALDEx2

RDA identified taxa contributing to the separation between the C and L groups, but this reflects contributions rather than direct statistical significance. Therefore, ALDEx2 was applied as a validation step. This analysis revealed taxa with significantly different relative abundances between the two groups and provided statistical support for several taxa highlighted by the RDA.

**Figure 5:**
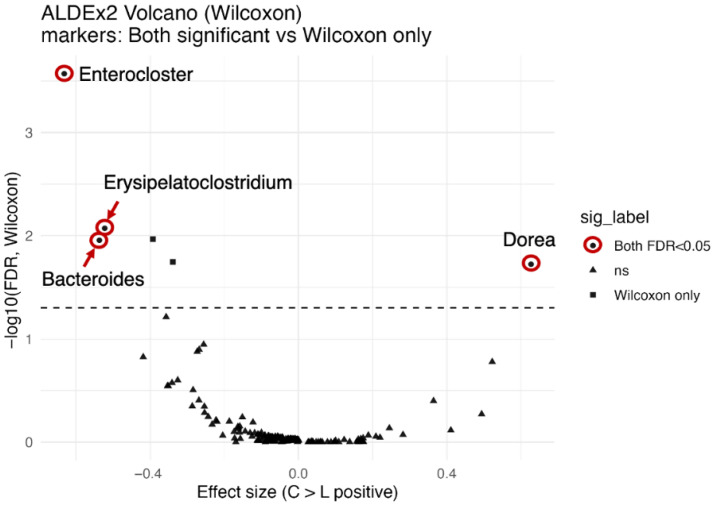
Volcano plots of differential abundance analysis results obtained via ALDEx2. The *x*-axis represents effect sizes, and the *y*-axis represents the −log10 of the false discovery rate (FDR)–adjusted *p* values from Wilcoxon rank-sum tests. Taxa were considered significant only if they had an FDR < 0.05 in both Wilcoxon and Welch’s *t* tests and an effect size > 0.5. Significant taxa differentiating the control and lean groups are highlighted. C(control): normal weight group; L(lean): underweight group. ANOVA-Like Differential Expression (version 2), ALDEx2.

The Bacteroides were significantly enriched in the lean group (L > C), with a clr mean difference of −0.94 and an effect size of −0.54. Both Wilcoxon and Welch’s tests supported these findings (Wilcoxon *p* < 0.001, FDR-adjusted *p* = 0.011; Welch *p* < 0.001, FDR-adjusted *p* = 0.013) (Figure 5, Table 6).

*Erysipelatoclostridium* were also significantly enriched in the lean group (L > C) (mean difference −2.64, effect size −0.52), with significant results (Wilcoxon *p* < 0.001, FDR-adjusted *p* = 0.0085; Welch *p* < 0.001, FDR-adjusted *p* = 0.015) (Figure 5, Table 6).

*Enterocloster* had the strongest association with the lean group (mean difference −2.74, effect size −0.63), with highly significant results (Wilcoxon *p* < 0.001, FDR-adjusted *p* < 0.001; Welch *p* < 0.001, FDR-adjusted *p* = 0.013) (Figure 5, Table 6).

In contrast, The *Dorea* was significantly enriched in the control group (L < C) (mean difference 4.89, effect size 0.63), with highly significant results (Wilcoxon *p* < 0.001, FDR-adjusted *p* = 0.019; Welch *p* < 0.001, FDR-adjusted *p* = 0.024). Thus, as consistent with the results of RDA, *Bacteroides*, *Enterocloster*, *Erysipelatoclostridium* were enriched in the underweight group (L > C), while *Dorea* was enriched in the normal weight group (L < C).

## 4. Discussion

Factors such as dietary habits and changes in the gut microbiota are considered to contribute to underweight in young women, but the actual situation in Japan remains unclear. In this study, we explored the differences between underweight and normal-weight young women by assessing their dietary habits and the diversity indices of their gut microbiota. Our analysis revealed no notable differences in the alpha and beta diversities of the dietary patterns; however, in contrast, the alpha and beta diversities (NMDS, RDA) of the gut microbiota significantly differed between the two groups. RDA further revealed that *Bacteroides*, *Bifidobacterium*, *Enterocloster*, and *Erysipelatoclostridium* were enriched within the underweight group, whereas *Fusicatenibacter*, *Agathobacter*, *Dorea*, and *Prevotella* were enriched within the normal-weight group. Moreover, AldEx2 analysis confirmed that *Bacteroides*, *Enterocloster*, and *Erysipelatoclostridium* were significantly more prevalent in the underweight group, whereas *Dorea* was more abundant in the normal-weight group. Further comprehensive research is necessary to elucidate these findings.

The number of the 10 food groups a person eats is an indicator of dietary diversity used in Japan [9,24,25,26]. Since the distributions of BMI and the Shannon index were bell shaped and peaked at approximately 18.5, this range may be the healthiest for young women, but further studies with more cases are needed. In other words, it is necessary to examine the relationships between the alpha diversity of dietary patterns and BMI by age group and sex across a wider range of ages. The reason for this is that when balanced meals are considered, the BMI range with the highest diversity is likely to represent the healthiest eating habits. While dietary pattern diversity was not related to BMI, components such as protein and dietary fiber, which are abundant in all 10 food groups, were strongly correlated with BMI. The alpha diversity of the 10-food-group dietary pattern allows for a very simple estimation of protein and dietary fiber intake, with meat, fish, eggs, dairy products, and soy belonging to the protein group and vegetables, seaweeds, fruits, and tubers belonging to the dietary fiber group. The alpha diversity of the diet may be a simple indicator for assessing dietary balance.

In this study, the alpha diversities of the gut microbiota in the underweight groups were lower than those in the normal groups. Lower values of the Shannon, Pielou, and Simpson’s diversity (1−D) indices consistently indicate reduced microbial diversity. Consistent with these findings, a systematic review on gut microbiome composition and stunting in children under five years of age reported that the alpha diversity of undernourished children is low [27,28,29]; however, some studies reported a decrease in alpha diversity, whereas others—including meta-analyses and reviews—have suggested that diversity is maintained or even increased in individuals with low body weight [30,31,32,33,34]. While some reports have documented a decrease in gut microbiota alpha diversity in children with malnutrition (poor nutrition/wasting and growth impairment), findings regarding extreme malnutrition in adolescents and adults (e.g., anorexia nervosa) have been inconsistent. In this study, 80% of the participants were 20−30 years old, which may have contributed to the consistent results, but our study cohort also included examples of individuals with high Shannon indices despite severe undernutrition. These results suggest that various factors, such as age, BMI, and region of residence, are associated with the diversity of the gut microbiota and that there may still be factors that have not yet been considered.

In this study, RDA revealed that dietary group assignment explained only a modest proportion of the overall variance in the gut microbiota (adjusted R^2^ = 0.032). Nevertheless, the observed separation between groups was statistically significant, indicating that even small shifts in community structure can be consistently captured when constrained ordination methods are used. These findings are consistent with previous reports demonstrating that host factors such as diet, age, or sex often explain only a fraction of the total microbial variation, with the majority attributable to interindividual differences and unmeasured environmental influences [35,36,37,38].

The taxa contributing most strongly to RDA1 included *Fusicatenibacter*, *Agathobacter*, and *Dorea*, which have been previously associated with fiber fermentation and short-chain fatty acid production. Conversely, *Bacteroides*, *Bifidobacterium*, *Erysipelatoclostridium* and *Enterocloster* were negatively associated with this axis, suggesting distinct ecological preferences between the groups. AldEx2 analysis also revealed that Dorea was relatively low and that *Bacteroides*, *Bifidobacterium*, *Erysipelatoclostridium* and *Enterocloster* were relatively highly abundant in the lean group. Stunting is associated with an abundance of pathobionts that can drive inflammation, such as *Escherichia*, *Shigella* and *Campylobacter*, and a reduction in butyrate producers, such as *Faecalibacterium*, *Megasphera*, and *Blautia*, and increased *Ruminoccoccus* [39]. Another review reported that malnutrition promotes a shift in the gut microbiome (increase in *Bacteroides*, *Clostridium*, and *Enterobacter*) [38]. These data are consistent with our data. These fluctuations in gut bacteria may explain the increase in inflammation. A decrease in Dorea leads to a reduced supply of anti-inflammatory SCFAs [40], whereas an increase in *Bacteroides* and *Erysipelatoclostridium* may be accompanied by inflammatory metabolites or changes in bile acid metabolism [41,42]. Additionally, an increase in *Bacteroides* and *Erysipelatoclostridium* suggests a high-fat, high-protein dietary pattern [43,44], and a decrease in *Dorea* may imply a reduction in dietary fiber intake, as members of this genus are recognized as short-chain fatty acid (SCFA) producers that utilize complex carbohydrates [45]. However, there were no differences between the two groups in terms of energy intake, protein intake, or dietary fiber intake, nor were there differences in the alpha or beta diversity of dietary patterns. Therefore, factors other than diet or abnormalities in the composition of the gut microbiota itself may contribute. Considering that underweight women have been reported to have decreased lymphocyte counts and impaired immune function, the increased presence of *Bacteroides*, *Enterocloster*, and *Erysipelatoclostridium* in the underweight group may reflect a greater burden of opportunistic bacteria contributing to intestinal inflammation and compromised mucosal barrier function. It is not clear whether this is a cause or a result, but further investigation is needed to determine whether individuals with low body fat percentages experience an increase in gut bacteria related to inflammation in underweight groups.

In this study, it was found that among underweight women aged 20–39, it was the diversity of gut microbiota patterns—not dietary diversity—that was reduced. While there are no studies comparing underweight and normal-weight women in Japan, research has been reported on patients with Anorexia Nervosa [46]. Fecal microbiota transplantation from AN patients to mice showed suppressed weight gain and changes in metabolism and neuronal gene expression, suggesting that gut microbiota may play a role in maintaining a low body weight. Germ-free mice were reconstituted with the microbiota of four patients with restricting-type AN (gAN mice) and four healthy control (gHC) individuals. Compared with gHC mice, gAN mice showed a decrease in body weight gain, concomitant with reduced food intake. This suggests that gut bacteria themselves may cause low body weight. Additionally, the Shannon index is positively correlated with the amount of short-chain fatty acids in feces [47]. Therefore, extrapolating from our findings, it is possible that reduced supply of short-chain fatty acids from the gut due to changes in the microbiota may contribute to weight loss. The fact that some people gain weight while others do not, even when eating the same food, might reflect the fermentative capacity of their gut bacteria. Accordingly, in addition to dietary fiber, the consumption of fermented foods may contribute to weight improvement in underweight women.

In this study, Japanese women aged 20 to 39 were surveyed, and their dietary fiber intake was found to be particularly low—about half of the recommended intake for Japanese people [48]. The dietary fiber intake of Japanese people, contrary to the common image of traditional Japanese cuisine, is actually quite low compared to other countries. According to the National Health and Nutrition Survey, fiber intake has decreased to about 70% of what it was around 1950, and currently remains at approximately 13–14 g per day [49]. This decline is considered to be mainly due to decreased consumption of grains (such as rice and barley) and an increased intake of animal-based foods, particularly meats, reflecting changes in dietary habits. Intake is especially low among women in their 20s (11.9 g), although it tends to increase with age [48]. Therefore, a practical target of 20 g per day has been recommended in Japan, while in the United States and Canada, the standard is set at 24 g. In comparisons between ethnic groups, Japanese people tend to have gut bacteria that utilize carbohydrates and amino acids more, whereas Westerners have more gut bacteria (archaea) that use hydrogen to produce methane [50]. These differences originate from variations in diet, and since the proportion of underweight women is lower in Western countries, there have been fewer studies targeting this population. Regarding the effects of age, dietary diversity (especially intake of vegetables, fruits, seaweed, etc.) increases as people get older, resulting in greater diversity of the gut microbiome. However, as people age, the number of confounding factors such as underlying health conditions also increases, making pure comparisons between weight groups more difficult. It was also considered that a low intake of dietary fiber may increase the impact of other environmental factors (such as other foods and nutrients) on the gut microbiota

The limitations of this study include that it is exploratory in nature and has a small sample size. Nevertheless, a clear separation of gut microbiota patterns was obtained, likely because the study was conducted on relatively young participants. In addition, matching for sex is also considered significant. Sex differences affect not only dietary preferences (eating patterns) but also eating behaviors such as eating speed and the number of chews, which are thought to influence the composition of the gut microbiota. Another limitation is that the BMI groups accounted for only approximately 4% of the observed variance. The factors influencing the gut microbiota can be broadly categorized as “diet,” “medication,” “age/sex,” “lifestyle habits,” and “diseases”. In this case, stress, sleep patterns, and the environment (residence, sanitation) clearly differed between the two groups, with the undernourished group being healthcare workers exposed to irregular work hours and stressful environments. For example, in shift workers such as doctors, nurses, pharmacists, and factory workers, night shifts may have an impact on their gut microbiota [51,52,53,54]. Given that controlling for all of these conditions simultaneously is extremely difficult, future studies may need to examine gut microbiota patterns in relatively young monozygotic twins in Japan. In such a design, the presence or absence of cohabitation could allow evaluation of the effects of differences in the dietary environment (primarily diet) on the gut microbiota. Finally, in this study, the FFQg was used; however, it is necessary to state clearly that there may be reporting errors in terms of energy and nutrient intake. For example, although the dietary fiber intake measured in this dietary frequency questionnaire was low, at around 10 g, our previous research using dietary record apps showed a fiber intake of 15.5 ± 5.1 g, while the FFQg and another commonly used Japanese food frequency questionnaire, the BDHQ (brief-type self-administered diet history questionnaire), showed 10.3 ± 3.9 g and 9.2 ± 4.0 g, respectively, displaying the same trend [55]. This may be due to the fact that dietary fiber intake in the FFQg and BDHQ do not include low-molecular-weight soluble fiber or resistant starch, as well as the possibility of underestimation due to self-reporting [55]. In addition, It is important to acknowledge that the food frequency questionnaire method may exhibit somewhat lower accuracy compared to other dietary assessment methods [56,57].

## 5. Conclusions

In this study, we demonstrated that gut microbiota patterns, rather than dietary patterns, differed significantly between normal and low-body-weight groups of young adult women, as evidenced by both alpha and beta diversity analyses. Furthermore, increases in *Bacteroides*, *Erysipelatoclostridium*, and *Enterocloster*, together with a decrease in *Dorea*, were observed in the low-body-weight group. These findings provide not only a foundation for developing clinical intervention strategies for underweight individuals but also important insights into the mechanisms underlying host–microbiota interactions. Examining the relationship between body fat percentage and gut microbiota patterns associated with lymphocyte count in underweight women may provide clues for clarifying the pathology of underweight women. Moreover, it is crucial to assess the effects of dietary interventions that incorporate both prebiotics (increased intake of dietary fiber) and probiotics (consumption of fermented foods) on the composition of gut microbiota and clinical parameters, such as body weight, in individuals who are underweight. At that time, it will be necessary to thoroughly examine what differences exist between the group that responds to such dietary therapy and the group that does not. This experiment may reveal that one cohort struggles to gain weight mainly due to changes in the gut microbiota, while another cohort finds probiotics ineffective because such changes have been caused by factors like intestinal stenosis. Finally, it will also be necessary to investigate how changes in the gut microbiota of underweight women affect their bodies in the future.

## Figures and Tables

**Table 1:** Participant characteristics.

Variable	Normal Weight Group (C)	Underweight Group (L)	*p*
	(n = 40)	(n = 40)	
Age, years	27.88 (4.65)	27.40 (4.49)	0.65
Weight, kg	53.84 (5.78)	43.36 (4.15)	**<0.001**
Height, cm	160.86 (6.05)	159.17 (5.10)	0.19
BMI, kg/m^2^	20.77 (1.41)	17.07 (0.97)	**<0.001**
Energy intake, kcal/d	1650.0 (666.6)	1564.0 (341.3)	0.48
Carbohydrate, g/d	212.73 (127.02)	205.48 (44.24)	0.74
Protein intake, g/d	55.21 (18.32)	53.90 (14.70)	0.73
Lipid intake, g/d	57.10 (15.52)	55.15 (14.38)	0.57
Dietary fiber, g/d	10.31 (4.54)	10.16 (3.33)	0.87

Comparison of age, BMI, and daily nutrient intake (energy, protein, fat, carbohydrate, and dietary fiber) between the normal weight (C: control) and underweight (L: lean) groups. The values are presented as the means (SDs). Bold letters indicates significant (*p* < 0.05).

**Table 2:** Alpha diversity indices of dietary and gut microbiota patterns.

Dietary Pattern				
Index	χ^2^ (*df* = 1)	*p*-Value	Cliff’s δ (95% CI)	Magnitude
Shannon index	1.06	0.30	−0.13 (−0.37, 0.12)	Negligible
Simpson’s diversity index	0.84	0.36	0.12 (−0.13, 0.35)	Negligible
Pielou index	0.37	0.54	−0.079 (−0.32, 0.18)	Negligible
**Gut Microbiota**				
**Index**	**χ^2^ (*df* = 1)**	***p*-Value**	**Cliff’s δ (95% CI)**	**Magnitude**
Shannon index	20.37	<0.001	0.59 (0.36, 0.75)	large
Simpson’s diversity index	18.09	<0.001	0.55 (0.32, 0.72)	large
Pielou index	26.4	<0.001	0.67 (0.46, 0.81)	large

Shannon, Simpson, and Pielou indices for dietary and gut microbiota patterns in the control (C, normal weight) and lean (L, underweight) groups. Cliff δ (95% CI) values are also shown. |δ| < 0.147: no effect; 0.147 ≤ |δ| < 0.33: small; 0.33 ≤ |δ| < 0.474: medium; and |δ| ≥ 0.474: large. A two-tailed *p* value < 0.05 was considered to indicate statistical significance. C: normal weight group; L: underweight group. *df*, degree of freedom.

**Table 3:** Correlations of Shannon diversity with BMI and nutrient intake.

	Shannon Index (Diet)		Shannon Index (Gut Microbiota)	
	ρ (95% CI)	*p* (Two-Tailed)	ρ (95% CI)	*p* (Two-Tailed)
BMI	−0.19 [−0.41, 0.029]	0.077	**0.49 [0.30, 0.65]**	**<0.0001**
Age	0.026 [−0.20, 0.25]	0.82	0.00042 [−0.22, 0.23]	0.97
Energy intake	0.28 [0.062, 0.47]	**0.013**	−0.066 [−0.28, 0.16]	0.56
Carbohydrate intake	0.20 [−0.017, 0.41]	0.07	−0.10 [−0.32, 0.12]	0.37
Protein intake	**0.49 [0.31, 0.64]**	**<0.0001**	−0.10 [−0.31, 0.2]	0.36
Lipid intake	0.31 [0.99, 0.50]	**0.0048**	0.027 [−0.19, 0.25]	0.81
Dietary fiber intake	**0.63 [0.47, 0.75]**	**<0.0001**	−0.13 [−0.35, 0.095]	0.24

Spearman’s rho (ρ) with 95% confidence intervals for associations between Shannon indices (dietary patterns and the gut microbiota) and BMI, age, and nutrient intake (energy, protein, fat, carbohydrate, and dietary fiber). Bold indicates significant (*p* < 0.05).

**Table 4:** Between-Group Differences in Dietary Patterns and the Gut Microbiota Assessed by PERMANOVA and PERMDISP.

Dietary Pattern							
Section	Metric	C (n = 40)	L (n = 40)	Contrast (Pair)	R^2^	F	*p*
PERMANOVA	Between-group composition	—	—	—	≈0	≈0	0.99
PERMDISP (overall)	Dispersion difference	—	—	—	—	0.44	0.52
PERMDISP (Tukey)	Pairwise	—	—	−0.013 (−0.050, 0.025)	—	—	0.51
Distance to centroid	Mean (BCa 95% CI)	0.14 (0.13–0.17)	0.13 (0.11–0.17)	—	—	—	—
Distance to centroid	Median (BCa 95% CI)	0.14 (0.11–0.15)	0.11 (0.087–0.13)	—	—	—	—
Dietary pattern							
**Gut Microbiome**							
**Section**	**Metric**	**C (n = 40)**	**L (n = 40)**	**Contrast (Pair)**	**R^2^**	**F**	***p***
PERMANOVA	Between-group composition	—	—	—	0.064	5.31	0.0001
PERMDISP (overall)	Dispersion difference	—	—	—	—	3.21	0.072
PERMDISP (Tukey)	Pairwise	—	—	0.025 (−0.0028, 0.053)	—	—	0.079
Distance to centroid	Mean (BCa 95% CI)	0.28 (0.27, 0.30)	0.31 (0.29, 0.33)	—	—	—	—
Distance to centroid	Median (BCa 95% CI)	0.27 (0.25, 0.31)	0.29 (0.27, 0.31)	—	—	—	—

Notes: PERMANOVA, permutational multivariate analysis of variance; PERMDISP, permutational analysis of multivariate dispersions. Distances were calculated via Bray–Curtis dissimilarity. The R^2^, F, and *p* values correspond to each test. Pairwise comparisons in PERMDISP were conducted via Tukey’s test. The distances to centroid values are shown as the means and medians with bias-corrected and accelerated (BCa) 95% confidence intervals obtained via 2000 bootstrap resamplings. For all the statistical analyses, two-tailed *p* values < 0.05 were considered to indicate statistical significance. C (control): normal weight group; L (lean): underweight group.

**Table 5:** Summary of Redundancy Analysis (RDA) Results.

Section	Metric	Value	Axis	Prop (Fraction)	Prop (%)	*p*	Term	F	*df*
Model summary	Total inertia	0.28							
Model summary	R^2^	0.045							
Model summary	Adjusted R^2^	0.032							
Model summary	Permutation F	3.65							
Model summary	Permutation *p*	0.0005							
Model summary	Permutations (N)								
		9999							
Model summary	Significant axes (*p* < 0.05)	1							
Model summary	Significant terms (*p* < 0.05)	1							
Model summary	List of significant terms	BMI group							
Model summary	R^2^	0.045							
Per-axis			RDA1	1	100.00%	0.0005			
Per-term						0.0005	BMI group	3.65	1

Notes: RDA, redundancy analysis. Total inertia indicates the total variance in the dataset. R^2^ and adjusted R^2^ denote the proportion of variance explained by the explanatory variables. Model significance was assessed via a permutation test with 9999 permutations. The per-axis results indicate the proportion of constrained variance explained by each significant axis (*p* < 0.05). The per-term results show the effect of each explanatory variable. Only the BMI group was significant in the present model. Abbreviation: Proportion, Prop; Degree of freedom, *df*.

**Table 6:** Differential abundance analysis of gut microbiota taxa between the control and lean groups via ALDEx2.

Feature	diff.btw	Effect	wi.ep	wi.eBH	we.ep	we.eBH
*Bacteroides*	−0.94	−0.54	<0.001	0.011	<0.001	0.013
*Enterocloster*	−2.74	−0.63	<0.001	<0.001	<0.001	0.013
*Erysipelatoclostridium*	−2.64	−0.52	<0.001	0.0085	<0.001	0.015
*Dorea*	4.89	0.63	<0.001	0.019	<0.001	0.024

Notes: Differential abundance analysis was performed via ALDEx2. A centered log-ratio (CLR) transformation with 2000 Monte Carlo samples from the Dirichlet distribution was applied to account for compositionality and technical variation. Group comparisons (Control vs. Lean) were tested via Welch’s *t* test (we) and the Wilcoxon rank-sum test (wi), with *p* values adjusted for multiple testing via the Benjamini–Hochberg method (eBH). “diff.btw” denotes the clr mean difference between groups, and “effect” indicates the standardized effect size. Taxa were considered significant only if they had an FDR < 0.05 according to both Wilcoxon and Welch’s tests. Wilcoxon test explained proportion, wi.ep; Welch’s t-test explained proportion, we.ep.

## Data Availability

All datasets generated and/or analyzed during the current study are not publicly available because they contain information that could compromise the privacy of research participants; however, they are available from the corresponding author upon reasonable request.

## Abbreviations

The following abbreviations are used in this manuscript:

PERMANOVA	permutational multivariate analysis of variance
PERMDISP	permutational analysis of multivariate dispersions
ALDEx2	ANOVA-Like Differential Expression (version 2)
NMDS	nonmetric multidimensional scaling
RDA	redundancy analysis
